# Evaluating clinical near-infrared surgical camera systems with a view to optimizing operator and computational signal analysis

**DOI:** 10.1117/1.JBO.28.3.035002

**Published:** 2023-03-29

**Authors:** Jeffrey Dalli, Abhinav Jindal, Gareth Gallagher, Jonathan P. Epperlein, Niall P. Hardy, Ra’ed Malallah, Kilian O’Donoghue, Padraig Cantillon-Murphy, Pól G. Mac Aonghusa, Ronan A. Cahill

**Affiliations:** a University College, UCD Centre for Precision Surgery, Dublin, Ireland; b IBM Research Europe, Dublin, Ireland; c University of Basrah, Physics Department, Faculty of Science, Basrah, Iraq; d University College Cork, School of Engineering, Cork, Ireland; e Tyndall National Institute, Cork, Ireland; f Mater Misericordiae University Hospital, Department of Surgery, Dublin, Ireland

**Keywords:** indocyanine green, fluorescence angiography, laparoscopy, anastomosis, near-infrared

## Abstract

**Significance:**

As clinical evidence on the colorectal application of indocyanine green (ICG) perfusion angiography accrues, there is also interest in computerizing decision support. However, user interpretation and software development may be impacted by system factors affecting the displayed near-infrared (NIR) signal.

**Aim:**

We aim to assess the impact of camera positioning on the displayed NIR signal across different open and laparoscopic camera systems.

**Approach:**

The effects of distance, movement, and target location (center versus periphery) on the displayed fluorescence signal of different systems were measured under electromagnetic stereotactic guidance from an ICG-albumin model and *in vivo* during surgery.

**Results:**

Systems displayed distinct fluorescence performances with variance apparent with scope optical lens configuration (0 deg versus 30 deg), movement, target positioning, and distance. Laparoscopic system readings fitted inverse square function distance-intensity curves with one device and demonstrated a direction dependent sigmoid curve. Laparoscopic cameras presented central targets as brighter than peripheral ones, and laparoscopes with angled optical lens configurations had a diminished field of view. One handheld open system also showed a distance-intensity relationship, whereas the other maintained a consistent signal despite distance, but both presented peripheral targets brighter than central ones.

**Conclusions:**

Optimal clinical use and signal computational development requires detailed appreciation of system behaviors.

## 1 Introduction

Near-infrared (NIR) camera systems enable the detection of photon emissions from fluorophores excited with specific wavelengths of light.^1^ Such systems have been medically deployed to detect the tricarbocyanine fluorophore indocyanine green (ICG) as an indicator of dynamic tissue perfusion. Once intravenously administered, ICG travels through the vasculature bound to blood proteins,^2^ where it has an excitation wavelength peak of 780 nm and a detected emission peak of 830 nm.^3^

Intra-operative ICG perfusion angiography (ICGPA) is used in both open and laparoscopic (“keyhole”) colorectal surgery to indicate colonic tissue circulation for diminishing malperfusion-related complications, such as anastomotic leakage.^4^^,^^5^ Similarly, this technology has been applied to reconstructive surgery with the scope of addressing ischaemia-related flap failures.^6^ Despite advances in camera systems and displays, interpretation of the fluorescence signal remains up to the surgical operator and is therefore prone to interpretation variability.^7^^,^^8^

Digitalization of the NIR signal has enabled quantification from still images and recordings of the dynamic display have been converted into fluorescence time series. Within research settings, temporal fluorescence intensity plots have been used to study the inflow and outflow of circulation. Curve features extracted from these plots have subsequently been correlated to postoperative outcomes.^9^ This work has also empowered the development of machine learning (ML) algorithms.^10^

Furthermore, investigational applications of quantitative analysis of tissue microperfusion have expanded this field to the characterization of tissue pathology (e.g., diagnosis of cancer in rectal polyps^11^) with opportunities in artificial intelligence powered cancer diagnosis.^12^^,^^13^ Also important in this is the inclusion of simultaneous white light imaging especially in laparoscopy to facilitate the corroboration of the fluorescence angiogram with the operative field (e.g., the prepared mesentery in colonic resection). For computational reasons, this view is needed to allow for tracking organs as they move (e.g., with respiration).

Although all clinical systems display an image of the fluorescence signal reflective of the tissue perfusion status under examination, their methods of excitation, sensing, and presentation differ due to both their inbuilt hardware and software components. These differences may impact operator-based interpretation and the development of computational tools. For instance, the distance^14^^–^^16^ and angulation^16^ from the tissue, as well as the positioning of the target in relation to the center of the screen,^17^ have been demonstrated to significantly impact the fluorescence signal. The relative importance of these physical variables may vary between systems.

### 1.1 Aims

This work assesses the impact of camera positioning on the displayed NIR signal across different commercially available and clinically used open and laparoscopic camera systems to better understand variability in this regard. Utilizing an electromagnetic stereotactic field generator (FG) with sensors (Aurora NDI, Canada) and software-based tracker-fluorescence quantification (IBM Research Ireland^18^), we generated test spatial and fluorescence data in both the clinical simulation model and operating theater setting.

We dynamically investigated the variations in the displayed fluorescence in relation to the camera-target distance, camera movement, and target positioning in a manner replicating clinical use in colonic perfusion assessment.

We also aimed to mathematically identify and recommend ideal parameters of use for bowel circulatory assessment relating to the fluorescence and optical performance of the camera systems tested.

In addition, we sought to demonstrate the ability to obtain these experimentally derived parameters in a theater setup during surgery, so they could be factored into clinically relevant computation.

## 2 Materials and Methods

### 2.1 Near-Infrared Systems

Six clinical NIR systems were assessed. Two are designed for open surgery, and four are laparoscopic systems with scopes featuring 0 and/or 30-deg lens configurations.

Two open systems were included: 1. Elevision, Medtronic, Ireland NIR system (EMO), 2. Spy-Phi, Stryker, USA system (SSO). The former is arm mounted, and the latter is handheld.

Four laparoscopic systems were included: 3. Pinpoint, Novadaq, Stryker, USA Laparoscopic system with both 0 (PNL0) and 30-deg (PNL30) lenses; 4. Elevision, Medtronic, Ireland Laparoscopic system with a 30-deg lens (EML30); 5. Synergy ID 4K, Arthrex, USA Laparoscopic system with 30-deg scope; and 6. Visera Elita II, Olympus, Japan Laparoscopic system with 30-deg scope.

Technical aspects of these systems were derived from user manuals, company correspondence, and publications and are shown in Table 1. To identify the optical filter(s) used in the NIR-specific (laparoscopic) scopes of each system, a broadband (360 to 2600 nm) light source (SLS201L, Thorlabs, United States) was directed through the lens channel of the scopes. At the output (opposite end), the wavelength information was captured and plotted (Optical Spectrum Analyzer: OSA, Thorlabs, United States) using a spectrometer (CCS200, Thorlabs, United States). The wavelength filter configuration of the scopes was inferred from the resultant spectral plots.

**Table 1:** Table demonstrating features of surgical camera systems tested in this experiment. Data from user manuals, communication with company representatives, or previously published assessments.^19^^–^^21^

		Open systems		Laparoscopic systems			
**Product**	**Company**	Medtronic (VisionSense) EMO	Stryker SSO	Stryker (Novadaq) PNL	Medtronic (VisionSense) EML	Arthrex	Olympus
	**System/device**	EleVision	AIM and SPY fluorescence	AIM and SPY fluorescence	Television	Synergy ID 4K	Visera Elite II
	**Camera**	Iridium	SPY-PHI	Pinpoint	Iridium	Synergy*UHD4* Broadband	CH-S200—XZ-EB
**Illumination/ Excitation**	**Light source type**	Laser	Laser	Laser	Laser	Laser	IR xenon bulb
	**Excitation wavelength (nm)**	785 or 805	805	805	785 or 805	785	710 to 790
	**Operation mode**	Continuous	20 pulses/sec (overlay mode) 40 pulses/sec (SPY mode)	20 pulses/sec	Continuous	Continuous	^*α*^
	**NIR source power output (max)**	3000 mW	2 mW	2 mW	3000 mW	^*α*^	300W bulb capacity
**Fluorescence Collection**	**Collection wavelength (nm)**	825 to 850^19^	830 (centered)^20^	825 to 850^19^	825 to 850^19^	840 (centered)	810 to 920
	**Sensor type**	CCD (×2)	CMOS	CMOS	CCD (×2)	CMOS (×4)	CMOS (×3)
	**Integration time**	Real time	Real time	Real time	Real time	Real time	Real time
	**Image overlay**	Yes	No	Yes	Yes	Yes	Yes
	**Variable gain setting**	Yes (two IR boost settings)	No	No	Yes (two IR boost settings)	No	No
**Optics**	**Working distance (cm)**	∼30	30	32^21^	∼30	^*α*^	^*α*^
	**Field of view (cm** ^**2**^ **)**	19 × 14	19 × 13^20^	19 × 14	19 × 14	^*α*^	^*α*^
	**Scope lens angle (Deg)**	30°	N/A	0 deg and 30 deg	30 deg	30 deg	30 deg
	**Scope filters** ^a^	N/A	N/A	720 to 820 nm notch (cut)	725-nm shortpass and 725-nm longpass	None present	640- to 820-nm notch (cut)

a Denotes experimentally acquired data and α denotes that the data was not available or obtainable through the previously mentioned sources.

### 2.2 Well Plate Design

A well plate was designed to reflect the size of the clinically relevant field of view for bowel perfusion assessment in the visual spectrum. Within a clinical trial (ethics approval 1/378/2092, ClinicalTrials.gov Identifier: NCT04220242), the length of bowel deemed clinically relevant by senior surgeons at the time of ICGPA during colon resection was measured with a sterile ruler. This included patients (n=6, 4:2 males:females, mean age 74 years) undergoing left and right (5:1) sided colonic resections (3:3 cancer:benign). Intraoperative measurements revealed a range of 12 to 15 cm, with the maximum length being used to inform the length of the clinically relevant field of view and the well plate dimensions.

**Fig. 1:**
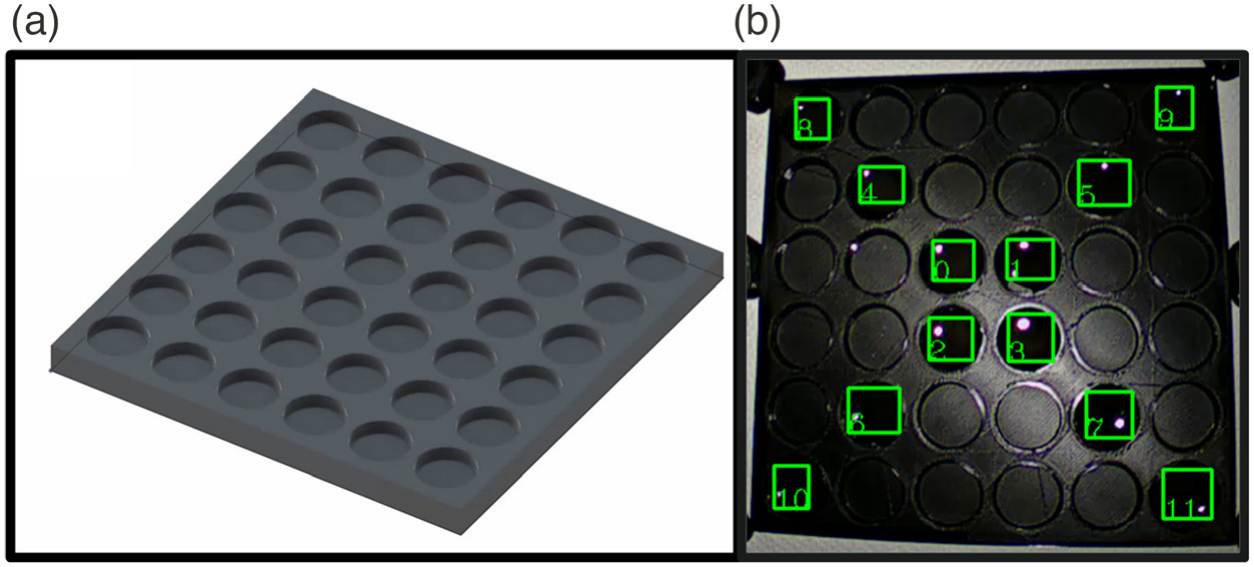
(a) 3D well plate design (6×6 wells, 15×5  cm), which was aliquoted with 0.4 ml of 0.0014  mg/ml ICG. (b) The green boxes correspond to the regions of interest (ROI) that the user annotated for fluorescence versus time tracking using the IBM bespoke tracker. This specific pattern of ROI annotation was used for peripheral fluorescence performance assessment. The inner ROIs (0 to 3) constitute zone 1, the middle ROIs (4 to 7) constitute zone 2, and the peripheral ROIs (8 to 11) constitute zone 3.

**Fig. 2:**
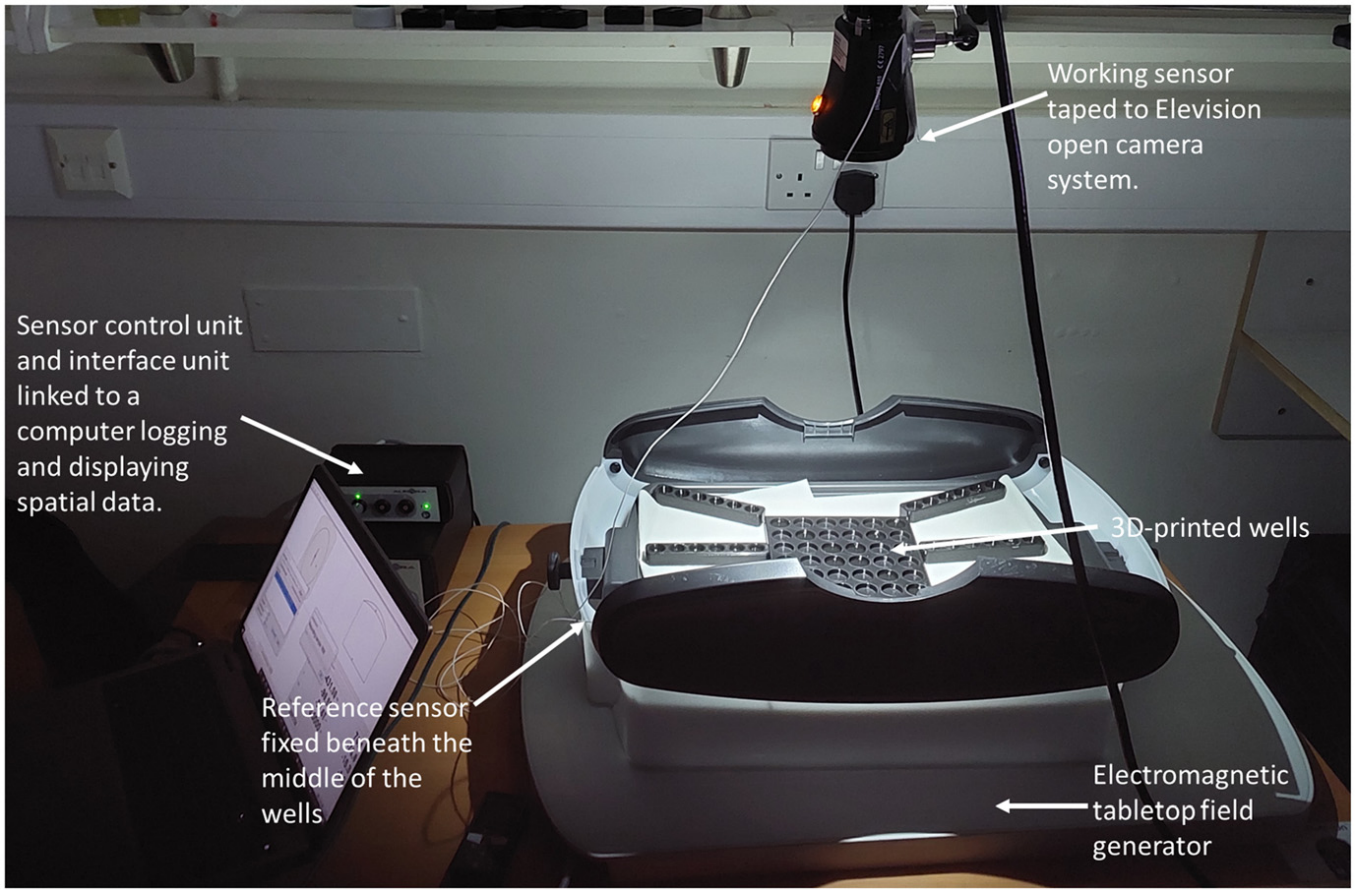
Experimental setup featuring an NIR system (Elevision, Medtronic, open system: EMO) with a sensor attached to the camera and a reference sensor underneath the wells, which are above the electromagnetic FG. A laptop records and displays spatial data form the sensor control and interface units.

Informed by the above, a square tile (15×15  cm) with equidistant wells (6×6 wells of 20 mm diameter and 5-mm depth) was then designed (AutoCAD Inventor, Autodesk, United States) and three-dimensional (3D)-printed in a matt low reflectivity material (black polylactic acid) (see Figs. 1 and 2). The 3D print was visually inspected for printing defects, and the dimensions were confirmed with vernier calipers. 3D print files for these well-plates are downloadable online.^22^

### 2.3 ICG Test Sample Setup

ICG (Verdye, Diagnostic Green, Germany) was diluted to 0.0014  mg/ml, representing a commonly used dose in clinical practice of 0.1  mg/kg (i.e., 7 mg ICG in a 70 kg ‘reference male’^23^ with a conventional total blood volume estimate of 70  ml/kg,^24^ i.e., diluted in 4.9 L) in human albumin solution (Flexbumin 200  g/L, Takeda, Ireland). 0.4 mL of this solution was aliquoted using medical syringes into each well of the 3D-printed well plate. The four innermost wells were termed “zone 1,” the wells immediately peripheral to the corners of these central wells were “zone 2,” and the outermost peripheral wells of the square were “zone 3” (Fig. 1).

### 2.4 Experimental Setup

The experiment was set up in a windowless room devoid of any natural illumination. An electromagnetic tabletop FG sensor system was positioned below the test site containing the ICG well plate to enable tracking of the real-time positioning of microsensors in 3D space including their orientation and angle via the emission of low intensity varying electromagnetic signals. This system is typically embedded into original equipment manufacturer medical instruments in image-guided surgery or methods in which position visualization and 3D tracking applications are applicable (e.g., interventional radiology^25^^,^^26^).

A reference sensor was fixed below the center of the 3D-printed wells (origin). These were in turn placed on the open base of a laparoscopic simulator box as the measurement volume of the FG is within the range of 120 to 600 mm above its top tangential surface. A second sensor (reference) was mounted to the tip of the scope/camera under assessment (see Fig. 2). The scope/camera (reference) sensor position was tracked with respect to the well plate sensor (origin), allowing for direct calculation of the 3D coordinate distance at any time stamp during the experiment from the x, y, and z coordinates via the following equation:

$$\text{Distance}=\sqrt{(x_{\text{scope}}-x_{\mathrm{ref}}{)}^{2}+{\left(y_{\text{scope}}-y_{\mathrm{ref}}\right)}^{2}+{\left(z_{\text{scope}}-z_{\mathrm{ref}}\right)}^{2}}.$$ (1)

Equation (1) is the distance computation equation.

Distance verification and testing beyond the vertical range of the FG (reported at 48 cm but experimentally set at 45 cm from the sensor) was carried out using a laser distance meter (PLR 30C, Bosch, Germany).

### 2.5 Initial System Screening

Following early technical assessments, laparoscopic camera systems incompatible with the tracking and quantification software were excluded. For the laparoscopic systems, compatibility was defined as the ability to track the target wells in zone 1 and quantify the fluorescence signal dynamically. For the open systems, only static tracking and quantification were possible as the EMO camera is attached to a flexible arm that impedes smooth movement, and these systems are clinically used at a fixed distance.

### 2.6 System Assessment

Systems were assessed as per manufacturer guidance with default settings applied. Specifically, for EML30 and EMO, the software boosting capability was locked and fixed at 99% with “default” settings otherwise to allow for quantification. The open systems were assessed statically with episodic movement to alter their distance from the wells. The laparoscopic NIR systems were assessed in three ways: statically at set distances (with only episodic movement to adjust the distance), continuous slow movement, and continuous fast movement of the scope tip toward and away from the filled ICG wells in a defined sequence. Between each of these tests and for each camera, a fresh mixture of ICG-albumin was prepared to minimize the effect of temporal chemical effects.

During testing, videos from the NIR systems and the stereotactic data from the electromagnetic FG were simultaneously recorded. The scopes of each system (with their mounted sensor) were manually tapped to induce a tracking artifact during the recordings for later (*post hoc*) chronological synchronization of the fluorescence intensity data with the spatial data (Microsoft 365 Excel, Microsoft, United States).

### 2.7 Fluorescence Signal Measurement

The centermost component within each ICG well was selected as the region of interest (ROI) (Fig. 1) in the videos obtained from each system during testing. The fluorescence intensities of these regions were analyzed using previously described software.^12^^,^^13^^,^^17^ This program tracks a user-annotated ROI, compensates for distance related ROI size deformation, and quantifies fluorescence intensity within it from the monochrome raw NIR feed in grayscale units (g.u.). By this means, variations in fluorescence intensity during camera testing were enumerated as a digital time series in comma-separated values file format.

Time-fluorescence data (22 frames per second (fps), 29.97 fps and 30 fps for EMO & EML30, PNL and SSO, respectively) were recorded by the software for zone 1 ROIs (in view throughout the testing). The fluorescence intensity in g.u. was subsequently averaged between the different ROIs within a given zone at each frame and plotted as a line graph. The stereotactic tracking data (40 fps), including distance versus time, were then synchronized to the fluorescence readings at the instance of manual tapping, followed by linear interpolation of the stereotactic data to the same values of time as the fluorescence data.

### 2.8 Distance-Intensity Relationships

To isolate the distance-fluorescence relationship, the time synchronized graphs were plotted as scatter plots (distance in mm versus fluorescence in g.u.). Where appropriate, inverse square functions [Eq. (2a)] and sigmoid curves [Eq. 2(b)] were fitted via regression (non-linear least squares) in python (scipy.optimize.curve_fit^27^). Goodness-of-fit was quantified via R^2^ (coefficients of determination) calculation.

$$y=\frac{A}{{\left(x-C\right)}^{2}}+B,$$ (2a)

$$y=\frac{A}{1\;+\;e^{B(x-C)}}+D.$$ (2b)

Equation 2(a) is the inverse square function, and Eq. 2(b) is the sigmoid function.

### 2.9 Ideal Fluorescence Distance

We developed a methodology for extracting an ideal fluorescence distance (IFD) from distance-intensity relationships. This metric is a distance that is close enough to avoid issues of low intensity and low signal to noise ratio (SNR) while far enough to avoid saturation and sharp changes in intensity with small changes in distance.

**Fig. 3:**
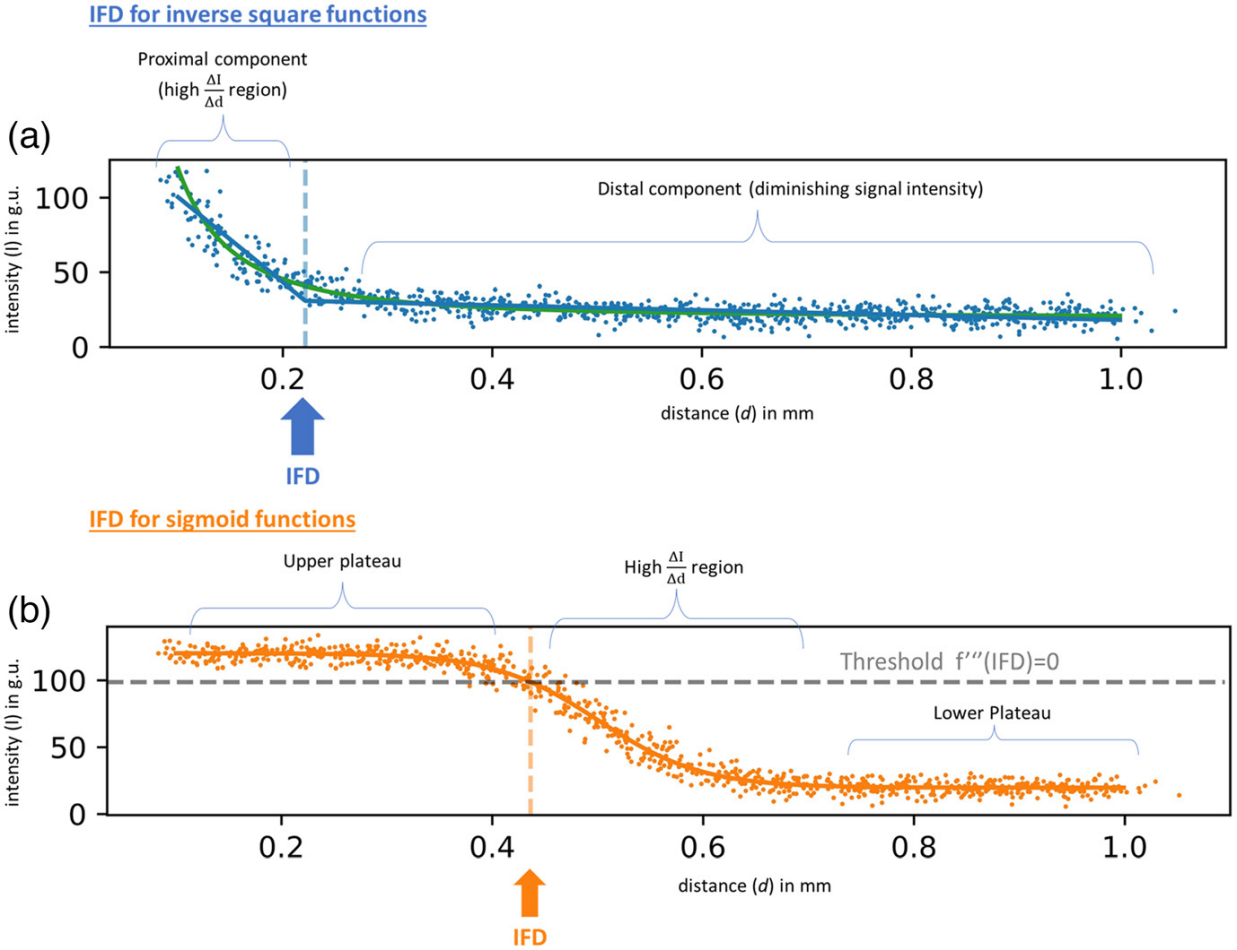
Using synthetic data, we illustrate the calculation of the IFD (ideal fluorescence distance) for two distinct patterns of intensity (in greyscale units, or g.u.) versus time (in seconds) observed in the real data. The scatter plot in panel (a) (blue points) was fitted with an inverse square function (green), and the scatter plot in panel (b) (orange points) was fitted with a sigmoid curve (orange). For inverse square fitted curves, the IFD was calculated by first fitting a “double line” (blue) using linear regression and recording the inflection point (blue dotted line) at which the slope changes from one line to the second. This point reflects a shift in gradient from the proximal, high $\left(\frac{\Delta I}{\Delta d}\right)$ gradient to the distal, lower gradient. For sigmoid fitted curves, the IFD (orange dotted line) was calculated using a previously reported threshold formula^28^ at 79% the total range of the sigmoid curve, which corresponds to the point at which the third derivative is zero (gray dotted line). This point reflects a shift from the proximal plateau of the intensity to a higher gradient region.

For distance-intensity relationships that followed an inverse square relationship, IFD was defined as the transition point between two components of the curve, the proximal and distal components (Fig. 3). The proximal component was ascribed to the region closest to the target where the signal intensity is highest and most prone to saturation and where the gradient of distance versus fluorescence is sharp. At the other end of the curve (the distal component), the signal suffers a diminished intensity and therefore a reduced SNR.

The IFD for inverse square functions was calculated as the transition point between these two regions via a double line method whereby two straight lines were also fitted via regression [Eq. (3) and Fig. 3].

$$y_{1}=ax+b\;y_{2}=cx+d\;y=\max(y_{1},y_{2})\;\mathrm{IFD}\;(\text{double line transition})=\frac{d-b}{a-c}.$$ (3)

Equation (3) is the double line method.

For sigmoid functions, the double line method could not be used to elicit the distance between shallow and sharp gradients. Utilizing a previously described methodology applied in human physiology,^28^ the IFD was calculated as the point at which the third derivative of the sigmoid function is zero (79% of range) [Eq. (4) and Fig. 3]. This allowed for the identification of the threshold beyond the plateau (which is prone to saturation and where no further distance-intensity changes take place). This distance also precedes the high gradient region of the sigmoid curve where, as above, changes in distance proportionally result in amplified changes in fluorescence.

$$y_{\text{sigmoid}}^{\prime \prime \prime }(\mathrm{IFD})=0\;\mathrm{IFD}\;(\text{sigmoid transition})=C-\frac{1.317}{B}.$$ (4)

Equation (4) is the sigmoid threshold equation,^28^ with C, B, and y taken from Eq. 2(b).

### 2.10 Ideal Optical Distance Measurement and Central Versus Peripheral Intensity Relationship Assessment

The IOD within this experimental setup was defined as the minimum distance required to visualize the full well plate (which was designed to reflect a clinically relevant length of bowel). This distance was extracted from the synchronized video and stereotactic (FG) data.

A central versus peripheral intensity relationship (CPR) intensity assessment was carried out at the IOD. All 12 wells (in zones 1 to 3, see Fig. 1) were annotated as ROIs, tracked, and their fluorescence quantified over a sampling period of one second. Subsequently, intensities of the four wells in each zone were averaged, and the mean zone fluorescence in g.u. was statistically compared with the Kruskal-Wallis test (following the Shapiro-Wilk test for normality) and *post hoc* pairwise testing with Bonferroni correction in SPSS.

### 2.11 Signal to Noise Ratio

Within the time versus fluorescence intensity profiles of each experiment, the rolling average (${\left\langle I\right\rangle }_{i}$) and standard deviation ($\sigma_{i}$) of the tracked ROI intensities (I in g.u.) were calculated using a window size of 20 data points [Eqs. (5) and (6)]. The SNR was calculated as the ratio of these two quantities in each window and was subsequently averaged across the given time series profile, with N being the total number of data points in a given time series [Eq. (7)^29^].

$${\left\langle I\right\rangle }_{i}=\frac{1}{20}\overset{i+20}{\underset{j=i}{\sum }}I_{j}.$$ (5)

Equation (5) is the rolling average.

$$\sigma_{i}=\sqrt{\frac{\overset{i+20}{\underset{j=i}{\sum }}{\left(I_{j}-{\left\langle I\right\rangle }_{j}\right)}^{2}}{20}}.$$ (6)

Equation (6) is the standard deviation of the intensity.

$${\mathrm{SNR}}_{i}=\frac{{\left\langle I\right\rangle }_{i}}{\sigma_{i}}\;\overset{¯}{\mathrm{SNR}}=\frac{1}{N}\overset{i=N}{\underset{i=1}{\sum }}{\mathrm{SNR}}_{i}.$$ (7)

Equation (7) is the SNR (rolling mean/standard deviation).

### 2.12 Velocity Effects

The velocity of movement (the distance moved divided by the time difference within one frame) was correlated to the frame ${\mathrm{SNR}}_{i}$ via Spearman’s correlation coefficient on SPSS (correlation<0.1 “negligible,” 0.1 to 0.39 “weak,” 0.4 to 0.69 “moderate,” 0.7 to 0.89 “strong,” >0.89 “very strong,”^30^ significance p<0.05) using SPSS (IBM, United States) version 27.

### 2.13 Angular Effects

Laparoscopic cameras are supplied in two optical lens configurations, with a 0-deg or 30-deg lens. Scopes with a 30-deg lens configuration require changes in scope angulation to maintain the ROI in view while varying the distance from the target. Thus, to assess the effect of the camera angulation related to the natural hand-held variation, only the 0-deg lens cameras for each system (PNL0, EMO, and SSO) were analyzed.

As the distance itself is a determinant of fluorescence intensity, corrections were needed to isolate the angular effects on fluorescence intensity. This was achieved by subtracting the previously fitted distance-intensity curves from the data to generate a residual [Eq. (8)]. The correlation between the residual intensity and the angle logged on the electromagnetic FG was calculated as Spearman’s correlation coefficient on SPSS.

Residual=Real fluorescence intensity−Predicted fluorescence intensity. (8)

Equation (8) is the residual fluorescence equation.

### 2.14 Clinical Testing

To demonstrate the clinical feasibility of this stereotactic setup, it was applied in a theater environment. This was carried out during rigid endoscopic examinations under anesthesia (EUA) of rectal lesions using PNL30 within the same clinical trial also investigating ICGPA-based cancer diagnosis. In this trial, the ICGPA of the lesions and surrounding healthy (control) mucosa was measured and compared, requiring the camera to visualize the necessary field of view to see both the tumor and control tissue.

Three setups (each in a different case) were used in the clinical testing. In each setup, the FG was fastened below the operating table’s cushioning, and a single sensor was attached to the tip of the scope (see Fig. 7). In one setup, two further sensors (one on the tumor and one on the control mucosa) were applied. In another setup, a sensor was used to touch the tumor prior to ICGPA using a surgical grasper. In the final setup, no additional sensors (other than the one on the camera) were used. The tumor distance was calculated by touching the tumor with the camera at the end of ICGPA (to avoid soiling the camera prior to ICGPA).

The ROIs were annotated postoperatively from the video recordings, tracked in white light, and quantified on the raw NIR feed using the above-mentioned tracker-quantifier, with distances extracted via the following equation:

$$\text{Distance}=\sqrt{{\left(x_{\text{scope}}-x_{\text{tumor}}\right)}^{2}+{\left(y_{\text{scope}}-y_{\text{tumor}}\right)}^{2}+{\left(z_{\text{scope}}-z_{\text{tumor}}\right)}^{2}}$$ (9)

Equation (9) is the clinically applied distance formula, where x, y, and $z_{\text{scope}/\text{tumor}}$ denote the coordinates in space for the scope tip and tumor, respectively.

## 3 Results

Experiments via the spectral analysis of passing a broadband light source through the laparoscopic system scope conduits are tabulated (see Table 1). The complete broadband source spectrum (360 to 2600 nm) plot obtained from testing system 5 indicates that no wavelength filters are integrated within its scope. System 6, PNL (0 and 30) scope plots the exhibited notch filter characteristics with wavelength cutting ranges of 640 to 820 and 720 to 820 nm, respectively. As the EML system incorporates a two-sensor configuration, its scopes contain two lens channels at the proximal end. Testing of both channels resulted in a 725-nm shortpass spectral plot for one channel and a a 725-nm longpass spectral plot for the other. From this, the functionality (white light versus NIR) of the channels was deduced.

Systems 5 and 6 did not present or store the white light feed separately, and this inhibited the software from tracking the target during motion purely on the NIR view, thus precluding them from further testing. Although SSO also did not present a separate white light feed, it was included as software tracking and quantification abilities sufficed for static testing. Furthermore, the comparable open system (EMO) limited dynamic testing anyway due to having mounting on a semi-rigid arm, i.e., dynamic motion testing was precluded for both open systems.

In general, the open systems (EMO and SSO) displayed a higher peak intensity (224.9 and 229.7 g.u.) than the laparoscopic systems (PNL 0, PNL30 105, 90.5, and EML30 73.8 g.u.).

**Fig. 4:**
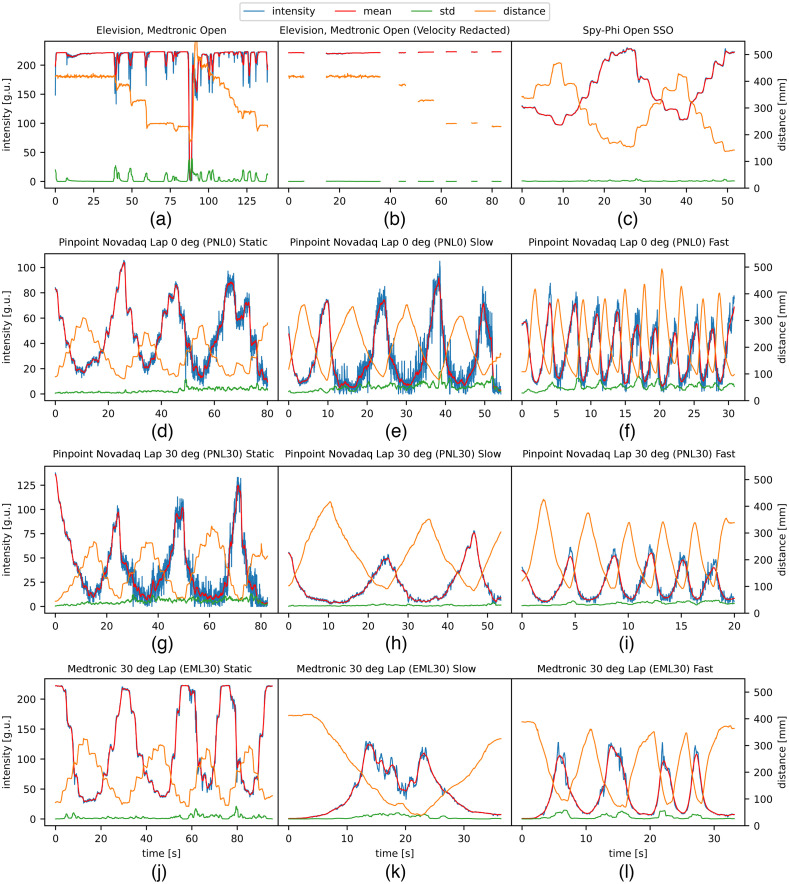
Compound plots displaying fluorescence intensity versus time plots (in seconds) for the open [panels (a)–(c)] and laparoscopic [panels (b)–(l)] NIR systems tested with different modes of camera movement (static, slow, or fast movement). The blue line is the intensity in grayscale units (g.u.) displayed on the far left axis for every panel. The red line is the rolling mean of intensity in g.u. displayed on the far left axis for every panel. The green line is the standard deviation of intensity in g.u. displayed on the far left axis for every panel. The orange line is the distance in mm displayed on the far right axis for every panel. The open camera systems [panels (a)–(c)] were only moved in the static fashion. The data for the EMO system with static movement are shown twice [panels (a) and (b)] with panel (b) showing the data after filtering out instances of repositioning the camera. The mean and standard deviation of the intensity were used to compute the SNR, which are not plotted (see Table 2).

**Fig. 5:**
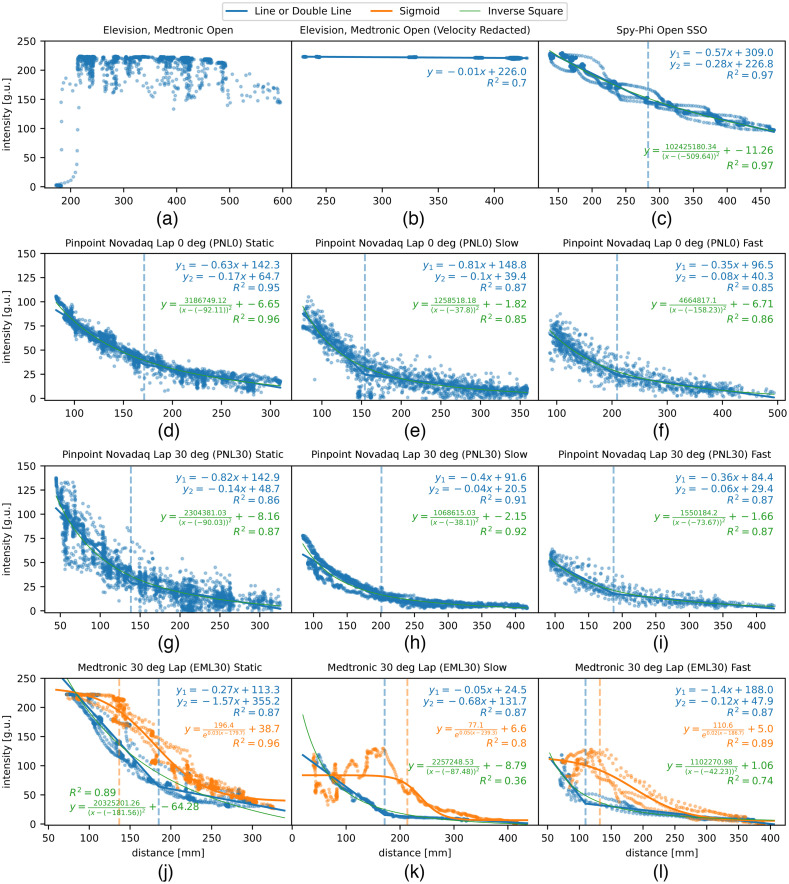
Compound plots displaying distance (mm) versus intensity in grayscale units (g.u.) scatter plots (blue and orange) with lines (blue), inverse square functions (green), sigmoid curves (orange), and their respective R^2^ values. The EMO system after velocity redaction [panel (b)] was fitted with a line (blue). The EML30 system [panels (j)–(l)] had distinct patterns when the camera was moving away from the fluorescent wells (blue dots) versus moving toward the wells (orange dots). The scatter plot where the camera approaches the well (orange dots) was fitted with a sigmoid curve (orange line). The data in the other systems were not split as such. All other scatter plots [panels (c)–(i) and part of (j)–(l)] were fitted with an inverse square function. In such cases, a “double line” was also fitted (blue line) to extract the IFD as described in Fig. 2.

Plotted lines for time-distance-intensity relationships (Fig. 4) and distance-intensity scatter plots (Fig. 5) revealed performance relationships. Intensity diminished with distance for all laparoscopic systems (EML30 and PNL), whereas for the open systems, it diminished for SSO but not EMO. Across all movement velocities, the fluorescence intensity range for the laparoscopic systems PNL0, PNL30, and EML30 were 105.6, 137.7, and 222.2 g.u., respectively. For the open systems, the intensity range for SSO was 132.7 g.u., but EMO showed no relationship between distance and intensity with a characteristically narrow intensity range of 4.23 g.u.

### 3.1 Open Camera Systems

#### 3.1.1 EMO

The stereotactic time-synchronized distance-intensity scatter plot fitted a flat line with fluorescence fluctuations chronologically related only to movement instances and not changes in distance. Redaction of the fluorescence during these instances of moving the camera of this system maintained a flat line even across large distance changes from 24 to 40 cm (y=−0.01x+226, $R^{2}=0.7$). Distances closer than 24 cm were inhibited by the system’s software. Testing beyond the range of the FG (determined by laser distance meter) revealed consistent performance up to 60 cm away with an IOD identified at 42.2 cm. Peripheral image interrogation also showed significantly raised peripheral intensity (zone 1: 221.45±2.11 versus zone 3: 223.10±3.42 g.u. p<0.001, see Table 3).

#### 3.1.2 SSO

This open handheld system revealed inverse square distance-intensity curves and demonstrated an IOD at 33.4 cm, with a maximal fluorescence signal at 15.5 cm. Zone 2 image signals were brighter than both the periphery and central zones (zone 2: 140.86±7.47 g.u. versus both zone 3: 121.68±15.06, zone 1: 114.81±8.67 g.u., p<0.001 for both). The double line calculation method of the Spy Phi open system showed an IFD of 28.3 cm.

### 3.2 Laparoscopic Camera Systems

Distance-intensity plots for laparoscopic systems (EML30, PNL0, and PNL30) revealed inverse square curves (see Fig. 5). Significant CPR effects were also noted for all systems (Table 3).

#### 3.2.1 PNL

The Pinpoint system (PNL0 and PNL30) curves fitted to R^2^ values of 0.86 to 0.95 (across static, slow, and fast). The 0-deg (PNL0) had an IOD at 14 cm as opposed to 24.7 cm for the 30-deg (PNL30), and the fluorescence signal was maximal at 10 and 8.27 cm for PNL30 and PNL0, respectively. The double line method displayed a range of IFD (across movement velocities) of 13.9 to 20.1 cm for the 30-deg and 15.4 to 20.9 cm for the 0-deg systems. The peripheral assessment revealed significantly diminished peripheral intensities with discrepancies between central zone 1 to the peripheral zone 3 (0 deg 21.01±4.67 versus 15.51±9.69 g.u. and 30-deg 9.44±1.45 vs. 8.37±1.95 g.u., both p<0.001).

#### 3.2.2 EML30

Results revealed a diminishing fluorescence intensity with increasing the distance. Visual interpretation of the scatter plot showed two distinct relationships. Splitting the fluorescence intensity values by direction, i.e., toward and away from the target, revealed a sigmoid fitting curve apparent on moving toward the well ($R^{2}=0.8$ to 0.96) and an inverse square function moving away ($R^{2}=0.36$ to 0.89) (see Fig. 5).

The inverse square curve (moving away) could be split with the double line method into two gradients with an IFD varying between 11 and 18.5 cm (across velocities). The curve approaching the wells showed a sigmoidal pattern requiring an alternative fit formula [Eq. 2(b)]. Fitting to a 79% threshold [Eq. (4)] (R^2^ between 0.8 and 0.96) to the sigmoid curve (approaching the wells) beyond the low distance plateau resulted in different IFD ranging from 13.2 to 21.4 cm.

The IOD was identified for the EML30 at 18.9 cm. The peripheral camera performance displayed significant intensity decreases progressing peripherally (21.35±6.14, 14.20±2.73, 9.66±3.23 g.u., p<0.001 across all pairwise combinations).

#### 3.2.3 SNR correlation

Correlating SNR with the velocity of movement revealed significant (both p<0.001) although negligible to weak correlation for EML30 (0.095) and SSO (0.154).

#### 3.2.4 Angular assessment

**Fig. 6:**
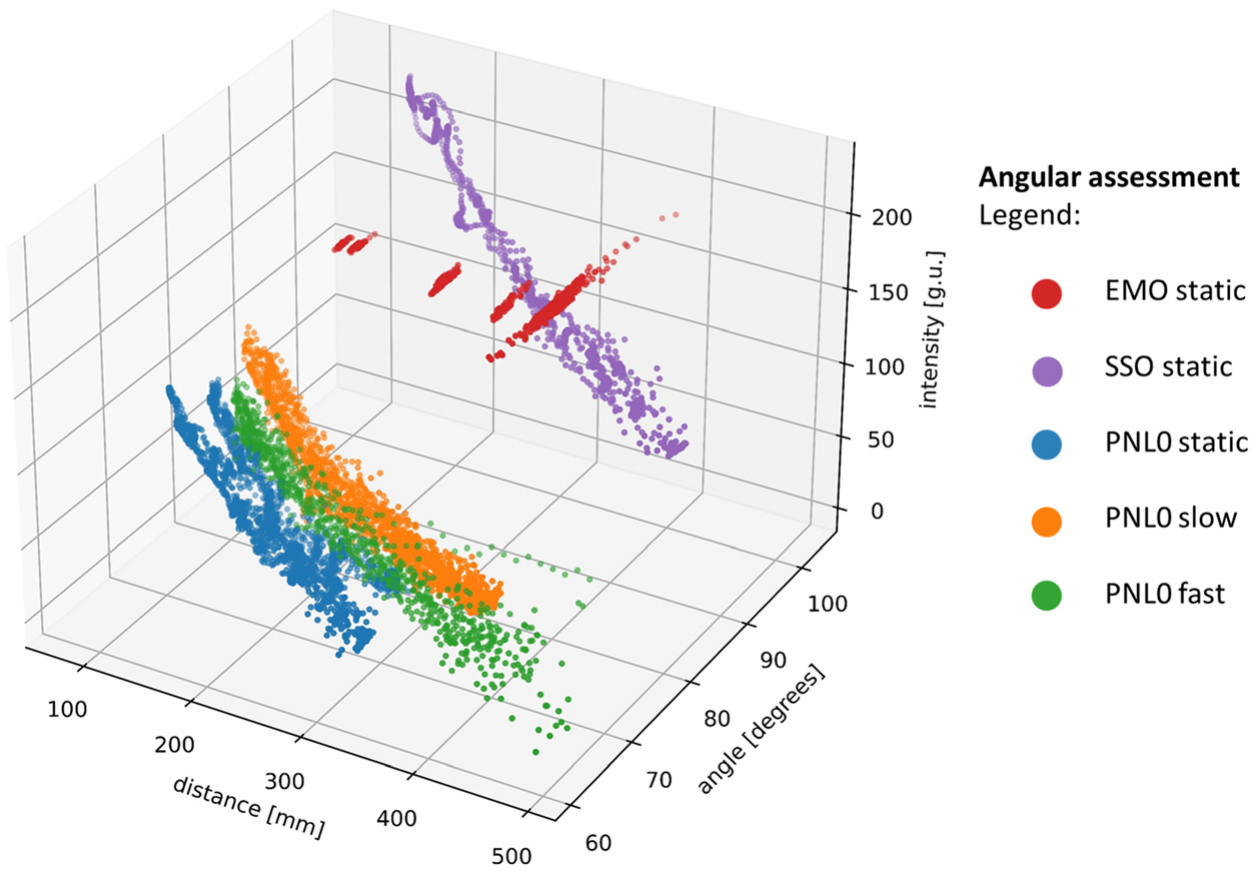
3D plot showing distance (mm), angle (degrees), and intensity (g.u.) to assess the effect of angulation on 0 deg camera systems. Red is the EMO system (only static camera movement) after periods of camera movement were redacted. Purple is the SSO system - only static camera movement was tested (including periods of movement). Blue is the PNL0 with static camera movement. Orange is the PNL0 system with slow camera movement. Green is the PNL0 system with fast camera movement.

Overall, the angular effects on intensity were negligible or weak. The range of angles detected (minimum to maximum) were as follows: PNL0 60.7 deg to 88.1 deg, EMO 66.7 deg to 94.5 deg and SSO 84.8 deg to 103.5 deg. For the PNL0, a significant correlation only existed during the static episodic assessment with a weak coefficient of 0.35 (p<0.001). However, pooling of all movements to include slow and fast movements resulted in negligible correlation (−0.07,p<0.001). EMO’s angular correlation was not significant, and the SSO system also displayed a negligible correlation (−0.09,p<0.001) (see Table 2 and Fig. 6).

**Table 2:** Table presenting results of testing of laparoscopic and open systems related to distance-intensity signal relationship. SNR denotes the signal to noise ratio.

				Mean velocity (mm/s)	Mean SNR	Fluorescence signal			Optical signal	Spearmann’s correlation: residual versus angle (p value)	
						IFD double line (and Sigmoid^b^) (mm)	Peak intensity (g.u)	Distance (mm)	IOD ideal optical distance (mm)		
**Open**	**Medtronic (EMO)**		Static	7.74	2649.9	N/A^a^	224.9	216.4	421.5	N/A	
			Movement redacted	2.73	3738.2					−0.05 (0.138)	
	**SPY-PHI (SSO)**		Static/episodic	21.10	342.4	283	229.7	155.56	334	−0.09 (<0.001*)	
**Laparoscopic**	**Novadaq**	**30 deg (PNL30)**	Static/episodic	20.17	13.6	138.5	90.5	100	247	N/A	
			Slow	25.99	21.8	200.9					
			Fast	151.97	7.4	187.1					
		**0 deg (PNL0)**	Static/episodic	17.12	28.3	171.2	105.0	82.65	140	−0.35 (< 0.001*)	−0.07 (< 0.001*)
			Slow	38.18	7.4	154.3				0.03 (0.283)	
			Fast	204.06	7.1	209.2				−0.04 (0.178)	
	**Medtronic 30 deg (EML30)**		Static/episodic	19.62	191.9	185.1 (136.7^b^)	73.8	40.33	189	N/A	
			Slow	20.72	22.7	171.5 (213.6^b^)					
			Fast	69.73	14.6	109.6 (132.1^b^)					

a Denotes that curve analysis was not carried out as the relationship was linear.

b Denotes calculation with the sigmoid thresholding formula [Eq. (4)].

* Denotes statistical significance, p<0.05.

**Table 3:** Table presenting results of testing of open and laparoscopic systems relating to central versus peripheral fluorescence differences.

			Center versus periphery assessment measuring fluorescence intensity (g.u.)						
			Zone			P values for Kruskal-Wallis and Pairwise (post hoc) tests			
			1	2	3	Kruskal-Wallis	1 versus 2	2 versus 3	3 versus 1
**Open**	**Medtronic (EMO)**		221.45 ± 2.11	221.34 ± 1.89	223.10 ± 3.42	< 0.001*	1	<0.001*	<0.001*
	**SPY-PHI (SSO)**		114.81 ± 8.67	140.86 ± 7.47	121.68 ± 15.06	<0.001*	< 0.001*	< 0.001*	0.328
**Laparoscopic**	**Novadaq**	**30 deg (PNL30)**	9.44 ± 1.45	8.64 ± 1.03	8.37 ± 1.95	< 0.001*	< 0.001*	1	< 0.001*
		**0 deg (PNL0)**	21.01 ± 4.67	15.26 ± 5.63	15.51 ± 9.69	< 0.001*	< 0.001*	0.924	< 0.001*
	**Medtronic 30 deg (EML30)**		21.35 ± 6.14	14.20 ± 2.73	9.66 ± 3.23	< 0.001*	< 0.001*	< 0.001*	< 0.001*

* Denotes statistical significance ascribed as p<0.05 (two tailed following Bonferroni correction for *post hoc* analysis). Zones 1 to 3 denote zones on 3D-printed well with zone 1 being the innermost and 3 the outermost.

#### 3.2.5 Operating theater trial

This setup was translated to an operating theater environment (n=3, m:f 1:2, mean age 71.3 years). These cases included transanal endoscopic assessment of rectal lesions prior to transanal endoscopic resection (n = 2 rectal carcinoma and a tubulovillous adenoma) and an assessment for treatment response post neoadjuvant therapy for rectal carcinoma. The operative workflow was not impeded by any of these methodologies.

**Fig. 7:**
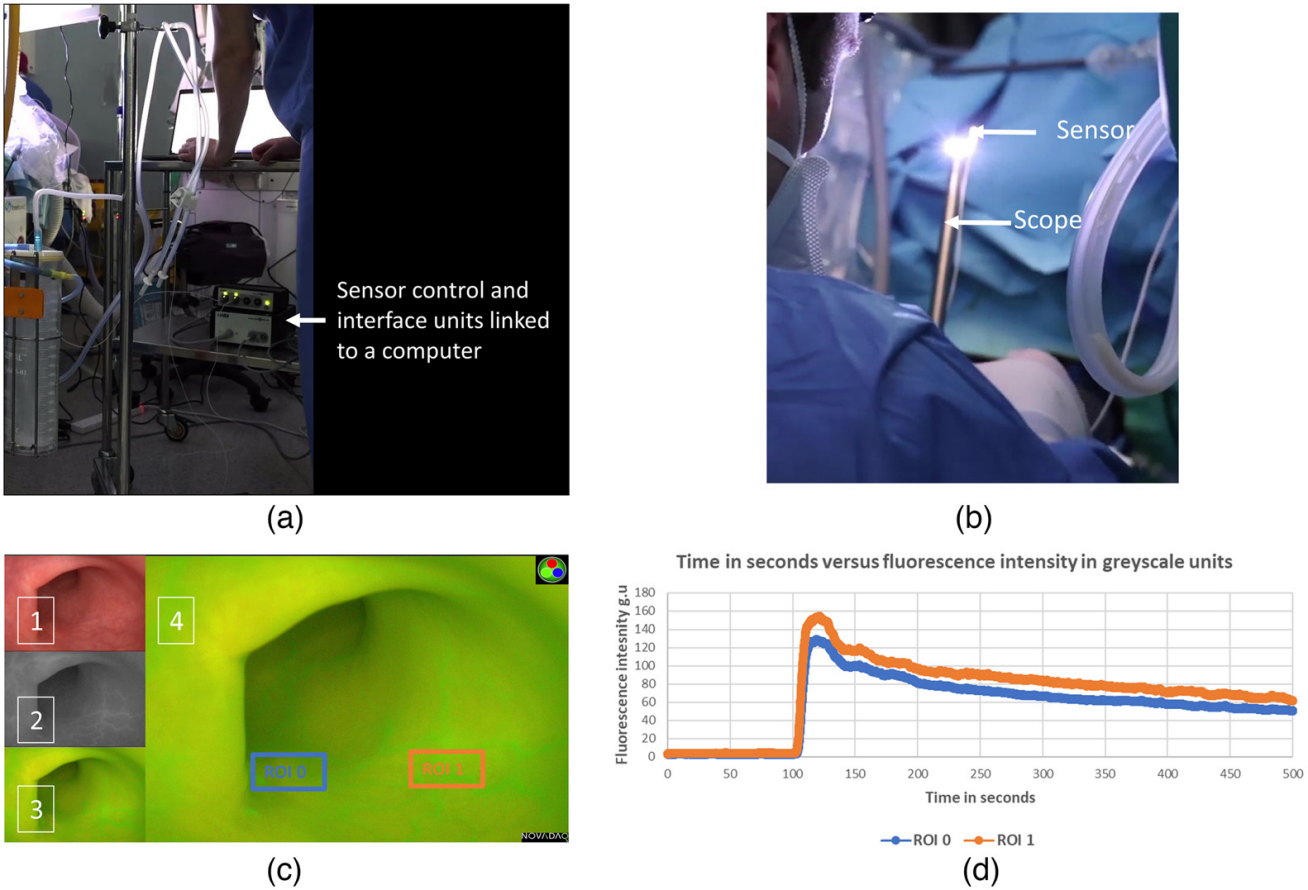
Composite image. (a) The operative setup with the sensor control and interface units set up on a trolley; (b) a sensor attached to a laparoscope (PNL30). (c) The displayed and recorded view of a rigid endoscopic examination under anesthesia for a patient with low rectal invasive adenocarcinoma post neoadjuvant treatment with a white light feed in panel (1), raw NIR feed in panel (2), and a hybrid image of the two in panel (3) and likewise in the main large image in panel (4). User annotated regions of interest (ROIs) for the scar of the preoperatively treated tumor (ROI 0) and normal mucosa as control (ROI 1) were tracked and their fluorescence quantified and plotted on the graph in panel (d) in time (seconds) versus intensity (g.u.) for the single sensor setup.

Utilizing three sensors (PNL30 camera, tumor and control), the tumor was visualized at 1.8 cm and control tissue at 7 cm from the camera. The dual sensor setup identified the tumor at 4.6 cm. The single sensor setup (camera only) computed a tumor to scope distance of 5.3 cm. Regions of surgical research interest (tumor and control) in the recorded endoscopic ICGPA colonic assessment were computationally annotated, tracked in white light, and their fluorescence quantified into a time series plot (see Fig. 7).

## 4 Discussion

This research details variations in fluorescence signal display between NIR camera systems and their scopes. The optical requirements of NIR fluorescence imaging require isolation of the emission wavelength range prior to sensing, necessitating noise (including excitation source reflectance from tissues) removal. Optical filters here were integrated into the rigid scopes in laparoscopic systems and into the cameras in open systems. Our investigational scope experiments illustrate the heterogeneous filtering strategies deployed to achieve the same goal (Table 1). Furthermore, even when using the same system and camera, the NIR signal varies by distance, velocity of movement, and across the screen (center vs. the periphery). These findings are important both for surgeons using different systems (for which efforts to standardize angiogram interpretation and training pathways to achieve this competence are still underway^31^) and for developers looking to advance computational methods for signal quantification for bowel perfusion. An inverse distance-intensity relationships was demonstrated for all systems except EMO.

At too great a distance, the signal weakens and thus results in diminished SNR. However, being too close predisposes it to signal saturation or results in sharp intensity fluctuations in relation to small changes in the camera distance. A previously reported methodology^14^ for IFD determination utilized linear actuators in vitro and utilized surgical rulers in vivo to determine the distance from the scope to preanastomotic colon. The authors identified the optimal zone for fluorescence detection as the inflection point of the high and low gradient portions (at near and far distances, respectively) of inverse square functions. However, this report^14^ did not mathematically define this location on the curve, and this methodology is not applicable to the sigmoid curves revealed in our work, which instead require threshold identification. Our reported methodology for assessment of IFD estimation offers a quantitatively determined distance that offers an option for protocolization and further research.

Computed IFDs varied with the rate of movement and method of determination (double-line versus sigmoid) and should be taken into consideration by users. For the sigmoid relationship assessment, the IFD was identified as the point at which the third derivative of the sigmoid function is zero, occurring at 79% of the range of the sigmoid curve.^28^ This methodology reproducibly identifies a point that surpasses the upper plateau region (which may signify signal saturation) and precedes the precipitous high gradient region of the sigmoid curve, which offers the same issues as the proximal component of the inverse square function (Fig. 3). Within this same methodology there also lies the opportunity of selecting an alternative distance at the tail end of the sigmoid curve prior to the lower plateau via a modification of Eq. (4) [Eq. (10)]. However, this alternative region occurs at lower fluorescence intensities and thus was felt to be an inferior option.

$$\mathrm{IFD}\;(\text{sigmoid alternative transition})=C+\frac{1.317}{B}.$$ (10)

Equation (10) is the alternative sigmoid threshold equation^28^ with C and B taken from Eq. 2(b).

Manual camera use would reflect static to slow movement (for example, when adjusting for respiratory movement or bowel peristalsis). Thus, from a fluorescence point of view, utilizing the camera for extracorporeal perfusion assessment at a distance between the computed IFD results for these two movements would seem reasonable. Second, one must also take into consideration the IOD that is identified as the minimum distance allowing for a field of view for assessing a clinically relevant length of bowel (see Table 2).

For example, the IFD data indicated that PNL0 should be used between 15.4 and 17.1 cm away from target for an ideal fluorescence performance. This also lies beyond the 14 cm IOD (i.e., minimum distance required to visualize a clinically relevant length of bowel). On the other hand, the 30-deg lens configured scope (PNL30) computed an IFD of 13.9 to 20 cm. However, its IOD exceeds this at 24.7 cm, precluding visualizing the full length of bowel at the IFD. This suggests that the PNL30 might be less appropriate for visualizing large lengths of bowel, e.g., in extracorporeal anastomosis than PNL0. This is despite the fact that scope lens angulation on the PNL30 allows the operator to hold the camera in a more natural position (as opposed to perpendicular to the operating table) and highlights the relevance of selecting different scope lens angle configurations. When applying this work to other clinical scenarios/specialties, one must keep in mind that, although the IFD is determined by the camera performance and thus is fixed per device, the IOD is determined by the intended use. For example, when assessing smaller regions, such as rectal polyps, the IOD would decrease.

At the IOD in this experimental setup, a CPR effect analysis that confirmed previously demonstrated in vivo peripheral signal hypoattenuation^17^ was significant with all laparoscopes (p<0.001). An operator should take this into account when examining for relative differences across the screen. The clinical implication of CPR is that tissue located centrally (e.g., bowel) on the screen appears brighter and relatively better perfused than tissue at the periphery. This disparity can potentially lead to confirmation bias, in which users make portions of the bowel appear relatively better perfused than they really are. Contrary to the laparoscopic systems and the inverse square law, in the open devices, the peripheries were not dimmer, and in fact, zone 2 (SSO) and zone 3 (EMO) were significantly brighter than zone 1. These results suggest that the equipment deploys strategies to defeat CPR. The impact of such “boosting,” whether hardware or software based on the quantification of ICG dynamic concentration, requires further investigation as their performance is complex and their closed box design limits compensatory software development.

A crucial requirement for fluorescence curve analysis relates to the peak fluorescence intensity and its chronology. The effect of CPR results in a relatively attenuated peak fluorescence at the peripheries. Although its impact on judgment has not been investigated, experts in the field have been demonstrated to unconsciously interpret these perfusions curve features^32^ as part of their decision-making process. Psychological research on visual fixation shows that observers perceive the center not necessarily at the geometric middle of the screen but in the lower third^33^ and tend to neglect brighter cues in favor of nearby less luminescent ones.^33^ This potentially further reinforces the interpretation of falsely attenuated peripherally placed tissues as regions of hypoperfusion.

Various methodologies to address CPR can be proposed. First, the quantified fluorescence curves can be normalized across the screen. This strategy removes variations in the peak intensity while maintaining the chronology of the flow, and in fact in vivo experimental evidence supports emphasis on time-based as opposed to fluorescence-based fluctuations.^9^^,^^34^ However, such work is focused on circulation assessment, and the fluorescence intensity signal is known to vary with ICG concentration and tissue thickness.^15^^,^^16^^,^^35^ Assessment of these criteria may be necessary for ICGPA tissue characterization based on dye accumulation (e.g., neoplastic lesion margination and depth assessment), and thus, signal normalization results in data loss. Mathematical flat field correction is well described in the field of microscopy;^36^^,^^37^ however, it requires a uniformly illuminated reference image for calibration. Dynamic CPR flat field correction is even more complex as it would require continuous calibration to compensate for potential changes in camera software effects over time.

Another issue that requires attention is signal saturation, i.e., detection or presentation of a signal beyond the dynamic range where further increases in the fluorescence are not appreciable visually or computationally. Our IFD calculation methodology seeks to avoid distances where saturation occurs. Saturation is less amenable to mathematical compensation than CPR as this lost apex data is realistically irreplaceable. Although some devices offer variable “smart” gain settings that automatically adjust to compensate for saturation, this fluctuating baseline fluorescence negates conventional curve analysis; hence, in this experiment these “smart” features were disabled under manufacturer direction where offered (e.g., EMO). ICG dosage also comes into play in this problem as higher doses are more likely to result in signal saturation. Clinical ICG dosage varies by usage, and reference dosages are now documented. In the literature documenting colorectal anastomosis assessment, this varies from 0.1−0.5  mg/kg.^38^ Consensus statements report a non-weight adjusted dose of 3 to 3.5 ml while identifying that dosage remains an ongoing research focus.^31^ For a 70 kg patient, 0.1  mg/kg results in 2.8 ml of ICG and thus is in the vicinity of the fixed dosage, too. Clinical dosing of ICG does not follow in vitro determination of ideal ICG concentrations. In vitro peak fluorescence was achieved at 30  μM (equating to 1.628  mg/kg);^39^ although less than the maximum daily allowance of 5  mg/kg,^40^ this greatly exceeds current clinical dosing.

An additional effect be taken into consideration is camera or scope angulation. In current clinical use with visual ICGPA interpretation, this is dictated by the necessity to optically visualize the target tissue. When one also takes into consideration the limited solid angle of view of the scope/camera, this allows for little leeway for angulation. However, the target tissue is inherently 3D (e.g., bowel, unlike our well plate), and although this may impact intestinal perfusion assessment, it may be especially important in, e.g., breast reconstruction in which the breast mound presents multiple angles of incidence for the scope. This phenomenon gains more importance in intraluminal applications of NIR systems in which the camera is closer to the ROI (e.g., polyp) and the range of viewing angles might be more pronounced due to viscus inaccessibility.

Within our dataset, the appreciable impact of angulation on 0-deg scopes (in which one aims to maintain a consistent perpendicular position, within the scope’s solid angle) suggests a weak correlation between angulation and fluorescence despite appreciable changes in angle. However, in this research, the goal of the operator was to maintain zone 1 in vision during camera movement. Due to the flat nature of the well design, scope angulation was not consciously challenged, and thus fluctuations are in fact only due to manual camera handling.

The distance-intensity-angle scatter plot for the EMO system displayed a distinct pattern (Fig. 6) that was likely due to the technical configuration of this specific system. This system displayed the narrowest range of fluorescence variation across all distances and angulations (4.23 g.u.), and this was reflected in the plot as changes in distance and angulation resulted in minimal changes in fluorescence intensity, not reflecting the inverse square law. This most likely reflects software/hardware compensation by the system in signal excitation, sensing, or *post hoc* display. Discontinuous clusters were appreciable, and these are due to the discrete testing distances and *post hoc* redaction of periods of movement due of to the semiflexible arm, which precludes continuous movement. There was also a visible increase in angle spread at greater distances. However, with Spearman’s correlation analysis (angle versus distance), the correlation factor was not significant, and these angular effects were likely related to the manual adjustment of the camera, which is both less accurate and of less impact at greater distances.

The implication of this system’s distinct performance is that the fluorescence signal does not change when these spatial parameters change, allowing users flexibility to compare angiograms even when these parameters have not been standardized. For example, in plastic surgery, free flap perfusion can be assessed with ICG at the flap design (while still connected to the original blood supply) and again once it has been anastomosed elsewhere.^41^ Having a reproducible fluorescence signal independent of spatial variations would allow for a computational perfusion comparison in different anatomical locations.

ICGPA is used in a variety of clinical contexts, and different IODs are relevant within different clinical scenarios. In this case, the IOD was determined for gastrointestinal perfusion assessment. Clinical interpretation techniques in this field can be broadly categorized into two distinct strategies: use of the system to assess perfusion as a potential tool for transection selection guidance (i.e., identification of the best perfused area for transection) or use of the system to confirm perfusion sufficiency where the transection region has already been selected (the most common current clinical use in colorectal surgery).

We determined clinically relevant bowel length ICGPA via the first strategy in which a large segment of bowel was assessed and, under fluorescence guidance, a transection region was selected as either best perfused or the best compromise between optimal perfusion and the remaining bowel length. This methodology is practical for visual interpretation as it allows for leeway for unexpected perfusion patterns as well as for postoperative, retrospective angiogram analysis and computational development of transection recommendation tools outside the theater environment, in a similar fashion to other quantitative endeavours.^9^

In a confirmatory role, the surgeon would have already selected a transection region and created an angiogram of a small segment of bowel, from which they would expect perfusion demarcation to manifest itself. If this focused region is suboptimally perfused, the operator can opt to trim the tissue proximally to theoretically have a better perfused bowel and not necessarily under fluorescence guidance. This would necessitate shorter distances (and less bowel visualized), diminishing the IOD. If the demarcation zone is missed, redosing and reassessing is an option; however, interpretation^32^ and quantification may be complicated by background fluorescence from incompletely cleared ICG (despite its short half-life of 3-5 minutes^32^). Other work by our group (under submission) has demonstrated that a protocol in which camera distance is arbitrarily standardized (without calculating the IOD) resulted in poorer interpretation consistency than *ad libitum* use.

The work is limited in being a bench model of a clinical use situation with only certain NIR systems being available for study (i.e., those in clinical use at our institution). The tracker software requires both white light and NIR display for robust motion defeating tracking, limiting us to dynamic tests of systems with such outputs. Such systems present these feeds as picture in picture or store both feeds separately. Although there are other systems in clinical use with this capability and others that lack it, the purpose here was not to provide an exhaustive analysis of all possible systems but to give indicative insights into system workings.

Our assessment on angular effects investigated changes related to manual use, and we have not sought to independently test the impact of a full range of angles on the camera performance. More detailed angular assessment would require the phantom to present multiple angles of incidence. Our well design is flat and not allowed to tilt, first because our target is liquid and second because large angles confound the distance peripheries, bringing them significantly closer or further way from the camera. 3D-printed solid phantoms^42^ may overcome such limitations. A further limitation of 3D printing is the potential printing inconsistencies and variations in well dimensions. Although manual post printing checks were carried out, this could be more thoroughly assessed by a well-by-well fluorescence comparison using a reference solution. Nonetheless, with some further adaptation, the 3D well plate (the design of which is now available) could be used as a reference tool to evaluate clinical NIR systems.

Another limitation of this work relates to handheld motion simulation with scope motion velocity being inconsistent during fast tests. Higher velocities were recorded during certain fast assessments (PNL0 and PNL30 20.4, and 15.2  cm/s) versus The EML30 (7  cm/s). Real-time operator wrist velocimetry would be a future consideration as speed was not presented on the stereotactic device dashboard. Motorized actuators setups^14^ would control for this but would not reflect current laparoscopic use. Increasing velocities of movement were paralleled by diminishing SNR, but correlation was negligible or weak. Furthermore, static assessment necessitates episodes of movement to vary the distance. Although longer periods of static readings could take place clinically, when assessing static solutions in vitro (as opposed to dynamically flowing blood *in vivo*), photobleaching must be considered^14^ as this may confound the result via time-related decreasing fluorescence intensity.

The single sensor operative setup demonstrated that stereotactic localization of the scope tip is feasible with referencing the scope by touching the tumor, relieving the need for a second sensor (e.g., clipped to the tumor, which would be prone to dislodgment due to sloughing and crossing the field of view). This setup did not obstruct the operative workflow, demonstrating that it could be feasibly integrated into procedures to support surgical stereotaxis, computational development, or device testing. A limitation of the clinical extrapolation is that this was only applied intraluminally and not extraluminally as would be the more common clinical application (in colorectal perfusion assessment). This was because intraluminal endoscopy does not require a sterile field, and therefore non-sterile sensors can be used on a single use basis. Sterilizable sensors could be integrated into the scope design or fitted inside sterile plastic covers (readily available for open NIR systems) if this might be helpful for quantification exercises and practice in the future. Analysis of the fluorescence performance endoluminally requires development a more complex benchtop model to factor for the enclosed, reflective features of the rectum and its mucosa.

In conclusion, fluorescence detection performance differs between systems and between various modes of operation in situations reflective of real-world use. The mechanisms of the systems as well as their use can impact the image being displayed, so care is needed in interpreting the images, most especially perhaps when considering computational analysis. Although such behaviors can be mathematically described and modeled, careful delineation is needed to best understand how the display is being achieved, and once understood, best practice and methods for computational quantification can be developed and applied.

## Code, data, and materials availability

The open-source code for the fluorescence tracker^18^ is accessible at https://github.com/IBM/optflow-region-tracker, and 3D print designs^22^ are available online at https://github.com/nolankucd/3D_Printed_well_plate. Data are available upon request.
